# Lipopeptides as the Antifungal and Antibacterial Agents: Applications in Food Safety and Therapeutics

**DOI:** 10.1155/2015/473050

**Published:** 2015-01-06

**Authors:** Khem Raj Meena, Shamsher S. Kanwar

**Affiliations:** Department of Biotechnology, Himachal Pradesh University, Summer Hill, Shimla 171 005, India

## Abstract

A lot of crops are destroyed by the phytopathogens such as fungi, bacteria, and yeast leading to economic losses to the farmers. Members of the *Bacillus* genus are considered as the factories for the production of biologically active molecules that are potential inhibitors of growth of phytopathogens. Plant diseases constitute an emerging threat to global food security. Many of the currently available antimicrobial agents for agriculture are highly toxic and nonbiodegradable and thus cause extended environmental pollution. Moreover, an increasing number of phytopathogens have developed resistance to antimicrobial agents. The lipopeptides have been tried as potent versatile weapons to deal with a variety of phytopathogens. All the three families of *Bacillus* lipopeptides, namely, Surfactins, Iturins and Fengycins, have been explored for their antagonistic activities towards a wide range of phytopathogens including bacteria, fungi, and oomycetes. Iturin and Fengycin have antifungal activities, while Surfactin has broad range of potent antibacterial activities and this has also been used as larvicidal agent. Interestingly, lipopeptides being the molecules of biological origin are environmentally acceptable.

## 1. Introduction

Extensive use of chemicals to control plant diseases has disturbed the ecological balance of microbes inhabiting soil leading to development of resistant strains of pathogens, groundwater contamination, and obvious health risks to humans. One of the biggest ecological challenges being faced by the microbiologists and plant pathologists in the future is the development of environmental friendly alternatives to the currently used chemical pesticides for combating a variety of crop diseases [1]. The continuous increasing drug resistance seen in bacteria has prompted a pressing need to find out some alternative antimicrobial molecules like lipopeptides to be used for clinical applications as well as in food preservation and dairy products [2]. Demand of lipopeptides is also surging due to their utility in human welfare, too. Lipopeptides were approved in the USA as antibiotics in the year 2003. Cubicin^R^ (Daptomycin), the first cyclic lipopeptide antibiotic was approved in USA by Food and Drug Administration (FDA) for the treatment of serious blood and skin infections caused by certain Gram-positive microorganisms [3]. These lipopeptides have projected peak annual US revenue of >US $1 billion and there use has been approved in more than 70 countries. Members of the* Bacillus *genus are considered as efficient microbial factories for the large scale production of such type of bioactive molecules [4, 5]. In the context of biological control of plant diseases, the three families of* Bacillus* lipopeptides, that is, Surfactin, Iturin, and Fengycin, were studied for their potent antagonistic activities against various phytopathogens [1]. Therefore, these compounds are widely considered as potential alternatives to the growing problem of resistance to the conventional antibiotics, fungal infections, and life-threatening diseases. Generally, bactericidal activity of the lipopeptide increases with the addition of a lipid moiety of appropriate length (typically C_10_–C_12_) and lipopeptides containing higher carbon atoms, such as 14 or 16, in lipid tail exhibit enhanced antifungal activity in addition to antibacterial activity [2]. Actinobacteria are species of the genus* Streptomyces* that has been reported to produce diverse antimicrobial lipopeptides with their applications in pharmaceutical industries [6]. Another lipopeptide (Polymyxin) interacts with an indispensable bacterial outer membrane component lipopolysaccharide (LPS). Polymyxin binds to LPS in Gram negative bacteria by electrostatic interaction by involving its N-terminal fatty acid tail that leads to its bactericidal action because of inhibition of synthesis of outer membrane [7]. Synthetic lipopeptides are widely used as vaccine adjuvants to enhance immune response, but bacterial derived recombinant lipopeptide, such as Lipo-Nter, is a novel adjuvant that can be used to induce superior antitumor effects as compared to a synthetic lipopeptide [8]. The principal representative of the lipopeptide family is Surfactin, which is produced by a bacterium* Bacillus subtilis*. Surfactin shows remarkable membrane-active and surface-interface properties resulting in a number of excellent biological activities, which are of great relevance in health care and biotechnology-based processes. These properties make Surfactin a potent candidate drug for the resolution of a number of global issues in medicine [9, 10], industry [11, 12], and environmental protection [13].

## 2. Types of Lipopeptides

Broadly there are three types of lipopeptides namely Surfactin, Iturin and Fengycin that are produced by various bacterial species.

### 2.1. Iturin

**Figure 1:**
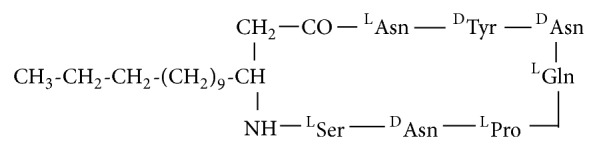
Cyclic structure of lipopeptide Iturin, containing seven amino acid residues attached to a 14-carbon chain indicates its amphiphilic nature. The amino acids involved in this structure are three D-amino acids (Tyr, Asn, and Asn) and the four L-amino acids (Pro, Ser, Asn, and Gln).

Amongst the three types of lipopeptides, Iturin is the small molecular mass of ~1.1 kDa. Iturin A consists of two major parts: a peptide part composed of 7 amino acid residues and 11-12 carbons hydrophobic tail (Figure 1). This structure clearly indicates an amphiphilic character of this compound, thus pointing towards the cellular membranes as the most probable site of their action [14]. Iturin lipopeptide is a cyclic peptide of 7 amino acids (heptapeptides) linked to a fatty acid (*β*-amino) chain that can vary from C-14 to C-17 carbon molecules. Such molecules are of great interest because of their biological and physicochemical properties, which can be exploited in food, oil, and pharmaceutical industries. All strains of* Bacillus subtilis* produce this family of lipopeptides.* Bacillus* sp. Iturin operon is 38–40 kb in size and consists of four open reading frames, namely,* ItuA*,* ItuB*,* ItuC,* and* ItuD* [15].

### 2.2. Surfactin

**Figure 2:**
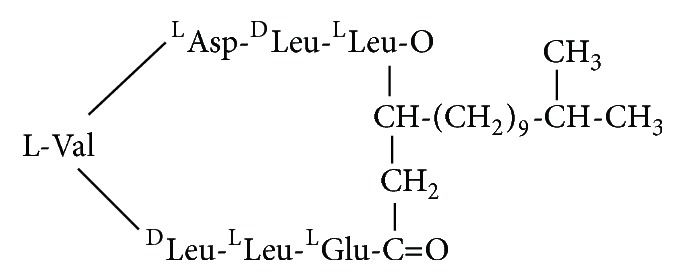
Heptapeptide cyclic structure of Surfactin, containing both hydrophobic and hydrophilic amino acids. The structure containing amino acids: two D-amino acids (Leu, Leu) and five L-amino acids (Val, Asp, Leu, Glu, and Leu), indicates its amphipathic nature.

Surfactin (~1.36 kDa) is an amphipathic cyclic lipoheptapeptide of Glu-Leu-Leu-Val-Asp-Leu-Leu (ELLVDLL) with the chiral sequence LLDLLDL interlinked with *β*-hydroxy fatty acid of the chain length of 12 to 16 carbon atoms to form a cyclic lactone ring (Figure 2) structure [16]. The same sequence of amino acids is found in a strain of* Bacillus* sp. namely AMS-H2O-1. The type of Surfactin may also vary according to the order of amino acids and the size of lipid portion [17]. Hydrophobic amino acids of Surfactin molecule are located at positions 2, 3, 4, 6 and 7 while the Glu and Asp residues are located at positions 1 and 5, respectively. Usually, Surfactin isoforms coexist in the cell as a mixture of several peptidic variants with a different aliphatic chain length [18]. The pattern of amino acids and *β*-hydroxy fatty acids in the Surfactin molecule depends not only on the producer bacterial strain but also on the type of culture conditions [16]. The *β*-turn may be formed by an intramolecular hydrogen bond, whereas the *β*-sheet may depend on an intermolecular hydrogen bond [19].

### 2.3. Fengycin

**Figure 3:**
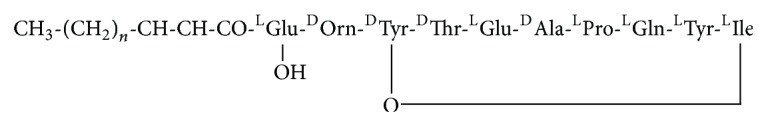
Primary cyclic structure of Fengycin A. Structure containing peptide chain of ten amino acids and a β-hydroxy fatty acid chain that can vary according to Fengycin isomer from C-14 to C-17 carbons. In the structure, the amino acids are six L-amino acids (Glu, Glu, Pro, Gln, Tyr, and Ile) and four D-amino acids (Tyr, Orn, and Thr, Ala).

Fengycin is a bioactive lipopeptide produced by several strains of* Bacillus subtilis*. It has antifungal activity against filamentous fungi [20]. It represents the third family of lipopeptides after the Surfactin and Iturin and is also called Plipastatin (Figure 3). These bioactive molecules are lipodecapeptides containing lactone ring in the *β*-hydroxy fatty acid chain that may be saturated or unsaturated. The structure of Fengycin contains a peptide chain of 10 amino acids linked to a fatty acid chain [21]. The length of the fatty acid chain can vary from C-14 to C-17 carbon atoms for Fengycins, thus giving different homologous compounds and isomers. Fengycins are cyclic decapeptide formed by lactonization [22]. The peptide portion of Fengycin lipopeptide consists of a decapeptide chain, of which 8 amino acids (Tyr, Thr, Glu, Ala, Pro, Gln, Tyr, and Ile) are involved in the formation of a peptide ring* via* lactone linkage between the side-chain phenolic–OH group of Tyr_3_ and C-terminal-COOH group of Ile_10_ [22]. Members of Fengycin family exhibit heterogeneity at the 6th position in peptide moiety as well as in chain length of *β*-hydroxy fatty acid, which varies from C-14 to C-17 carbons [23]. On the basis of variation at single amino acid at the 6th position in peptide ring, Fengycins have been classified in two classes, namely, Fengycin A and Fengycin B. Fengycin A contains Ala at position 6 which is replaced by Val in case of Fengycin B.

## 3. Lipopeptides-Based Biosurfactants

Lipopeptides as biosurfactants have been used in biomedical and pharmaceutical applications as antimicrobial, antimycoplasma, antiadhesive, and antitumor agents [41]. These biosurfactants from* Bacillus* spp. are renowned and most effective microbial surfactants [42]. These lipopeptides surfactants are environmental ecofriendly alternatives to synthetic surfactants. There has been an increasing interest to study the effect(s) of lipopeptide biosurfactants on human and animal cell lines. Some of the roles of these biosurfactants include their use as antiadhesive agents to pathogens, thus making them useful therapeutic, probiotic, and pharmaceutical agents [43].

## 4. Lipopeptides as Biocontrol Agents

Lipopeptides act as biocontrol agents because of their property of inhibition of growth of a variety of microorganisms including phytopathogens (Table 1).

## 5. Applications of Lipopeptides

**Figure 4:**
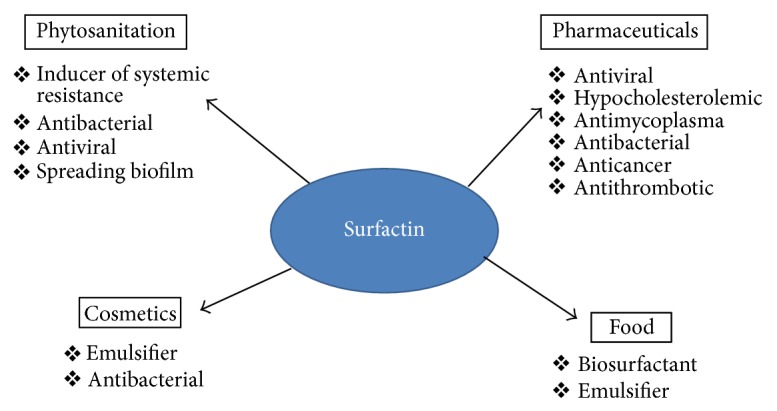
Broader applications of Surfactin in food and pharmaceutical industries. Applications are shown in different areas such as phytosanitation, pharmaceuticals, food, and cosmetics.

Out of the three lipopeptides (Iturin, Surfactin, and Fengycin), Surfactin has been preferentially considered for various commercial applications (Figure 4). Several recent reviews summarize the high interest in the use of biosurfactant for applications [44] in foods [12], environmental management [13, 45], biomedical fields [32], and cosmetics [46]. During its long history, Surfactin was first studied for its potential pharmaceutical applications (antibacterial, antitumor, and cholesterol lowering activities). The discovery at the end of 1990s of its antimycoplasma and antiviral properties leads to the proposal of its use to ensure the safety of biotechnological or pharmaceutical products. The presence of lipopeptides in fermented food products [47] was also considered for their applications in the food sector. Moreover, their ability to induce systemic resistance in plants and their use in the spreading of the bacterial cells leading to rhizosphere colonization could open new fields of applications for their use as promising phytopharmaceutical products.

### 5.1. Lipopeptides in Food Industry

The use(s) of lipopeptides as antimicrobial peptides/food preservatives are limited because of their inherent sensitivity to proteases. This sensitivity can be prevented by using peptides having cyclic ring-structure such as lipopeptides [48]. Lipopeptides in the food industry are well characterized in the terms of their antiadhesive, antimicrobial, antiviral, and antitumor activities, which ensure their position and important roles in the industries such as pharmaceutical and cosmetics [48]. In the food industry, lipopeptides can be used as emulsifiers in the processing of raw materials. In the baking industry, Surfactins are used to maintain the texture, stability, and volume and also to help in the emulsification of fat in order to control the aggregation of fat globules [48]. Recently, some lipopeptides isolated from bacterial group,* Enterobacteriaceae*, have been introduced into the food industry with their high emulsifying properties at enhanced viscosity at an acidic pH [48]. Often various food preservatives are used by food manufacturers during processing to avoid rapid food spoilage. Among biopreservatives, several antimicrobial compounds have been accepted till date. These compounds effectively control food poisoning microbes [49]. Sale values of food additives are growing continuously at a rate of about 2 to 3% annually. In terms of market increase, the most significant growth rates in food additives were observed for emulsifiers and hydrocolloids [50]. It is quite likely that lipopeptides, in the near future, will represent significant percentage of food additives in the market.

### 5.2. Biomedical and Therapeutic Applications of Surfactins and Iturins

Among several categories of biosurfactants, lipopeptides are particularly interesting because of their high surface activities and antibiotic potential against an array of phytopathogens. Surfactins can act as antiviral agents, antibiotics, antitumor agents, immunomodulators or specific toxins inhibitors (Table 2). Surfactin was found to be more efficient than Iturin A in modifying the* B. subtilis* surface hydrophobic character [51]. Conjugates of lipopeptide and T-cell epitopes also constituted effective adjuvants for the* in vitro* immunization of either human mononuclear cells or mouse B cells and resulted in an increased yield of antibody-secreting hybridoma.

### 5.3. Surfactin: An Antimycoplasma Agent

Mycoplasma is the smallest free-living organism and parasite of eukaryotic cells and is one of the major contaminants that affect mammalian tissue culture cells. Mycoplasmas are serious causative agents of diseases of both humans and animals, such as acute respiratory inflammation (including pneumonia), urogenital tract infections and AIDS [52, 53]. Treatment with antibiotics is the most effective procedure for eliminating or suppressing mycoplasma infection in the cell cultures. Surfactin is used commercially for curing of cell cultures and cleansing of biotechnological products of mycoplasma contamination [54]. In general, antibiotic therapies are successful in long lasting successful decontamination and do not show undesirable side effects/cytotoxic effects on eukaryotic cells [55, 56]. Surfactins have versatile bioactive properties with significant antimycoplasma activity [57]. Their disintegration is obviously due to the physicochemical interaction of the membrane-active Surfactin with the outer part of the lipid membrane bilayer, which causes permeability changes and at higher concentrations leads finally to disintegration of the mycoplasma membrane system by its detergent-like effect.

### 5.4. Cyclic Lipopeptides: Mosquito Larvicidal Agent(s)

Mosquitoes are blood feeding insects and serve as vectors for spreading human diseases such as malaria, yellow fever, dengue fever, encephalitis, West Nile fever, and lymphatic filariasis. The culture supernatant of a Surfactin-producing* Bacillus subtilis* strain was found to effectively kill the larval and pupal stages of mosquito species such as* Culex quinquefasciatus, Anopheles stephensi,* and* Aedes aegypti* [16]. As some biocontrol agents or insecticides are effective against mosquito pupae, this could be a good tool for application in malaria control programmes [58]. Further, growing public awareness about the environmental and human risk associated with chemical pesticides, emergence of pesticide resistant insect populations and rising prices of chemical pesticides has invariably stimulated the search for new eco-friendly vector control biological tools [59]. In this respect, several biological control agents have been tested in India and in many other parts of the world to evaluate their potential to control the mosquito vectors [60]. Toxins from certain strains of bacteria, such as* Bacillus thuringenesis* var.* israelensis* (Bti) and* B. sphaericues* (Bs), are shown to be highly effective against mosquito larvae at very low dosage and they are also safe to nontarget organisms [61]. However, the biolarvicide formulation from Bs strain is reported to be less effective against* Anopheles culicifacies* and hardly effective against* Aedes aegypti* [59]. A potential key strategy for delaying resistance to mosquitocidal proteins is to use a mixture of toxins that act at different targets within the insects [62].

### 5.5. Antiparasitic Activity of Surfactin

Microsporidia are defined as highly specialized fungi [63].* Nosema ceranae* is one of the etiologic agents of nosemosis, a worldwide disease [64]. Surfactin is considered as a molecule capable of reducing parasitosis development, acting either by direct exposure to spores or by its incorporation in the luminal of bee midgut [65]. Surfactin functions as a competitive inhibitor of NAD^+^ and an uncompetitive inhibitor of acetylated peptide. Surfactin was also found to be a potent inhibitor of intraerythrocytic growth of* P. falciparum in vitro* [66]. Surfactin can also be used as alternative treatment for nosemosis. When exposed to Surfactin, the spores of* Nosema ceranae*, the causative agent of parasitic infection in* Apis mellifera*, revealed a significant reduction in infectivity [65]. Moreover, when Surfactin is administered and is introduced into the digestive tract of a bee, it also leads to a reduction in parasitoids development [65].

### 5.6. Antiviral Activity of Surfactin

Surfactin is also active against several viruses, including the Semliki Forest virus,* Herpes simplex virus* (HSV-1 and HSV 2),* Simian immunodeficiency virus, Vesicular stomatitis virus, Feline calicivirus,* and the* Murine encephalomyocarditis virus.* The length of the carbon chain in cyclic Surfactin lipopeptide influences its capacity for viral inactivation [67]. The inactivation of enveloped viruses, especially herpes viruses and retroviruses by Surfactin, is significantly more efficient than that of nonenveloped viruses [16]. This suggests that the antiviral action of Surfactin is primarily due to the physicochemical interaction between the membrane active surfactant property of Surfactin and the virus lipid membrane [33]. One important factor for virus inactivation is the number of carbon atoms in the acyl chain of Surfactin. The capacity for virus inactivation increases with rising fatty acid hydrophobicity [16]. During the inactivation process of viruses, Surfactin permeates into the lipid bilayer thereby inducing complete disintegration of the envelope containing the viral proteins involved in virus adsorption and penetration to the target cells. Its absence accounts for the loss of viral infectivity [68]. Thus Surfactins have demonstrated antiviral activities [38]. It has also been observed that antimicrobial lipopeptides containing Surfactin inactivate cell-free viruses of the* Porcine parvovirus, Pseudo rabies virus, Bursal disease virus,* and* Newcastle disease virus* [69].

### 5.7. Antitumor Activity of Surfactin

Surfactin is a potent lipopeptide considered as a versatile bioactive molecule with antitumor activity [38]. Surfactin has been reported to show antitumor activity against Ehrlich's ascites carcinoma cells [35]. The effect of Surfactin as cytotoxic agent on the proliferation of human colon carcinoma cell lines such as HCT15 and HT29 [70] has also been reported. The inhibition of growth of transformed cells by Surfactin was due to the cell cycle arrest and induction of apoptosis* via* the suppression of cell survival regulating signals such as ERK and PI3 K/Akt [71]. The percentage of viable cells decreased with increasing Surfactin concentrations and exposure time that indicated its cytostatic/cytotoxic effect against breast cancer cell lines like T47D and MDA-MB-231 [72]. Another study revealed that Surfactin inhibits proliferation and also induces apoptosis of human breast MCF-7 cancer cells trough a ROS/JNK-mediated mitochondrial/caspase pathway in a dose-dependent manner [16, 73]. Surfactin generates the reactive oxygen species (ROS), which activate the mediator of survival and JNK and ERK1/2, which are the key regulators in apoptosis. These results showed that the action of Surfactin seems to be realized* via* two independent signaling mechanisms [10]. The induction of apoptotic cell death is an emerging strategy for the prevention as well as treatment of cancer.

### 5.8. Thrombolytic Activity of Surfactin

The plasminogen-plasmin system involves the dissolution of blood clots in a variety of pathological and physiological processes requiring proteolysis. Zymogen plasminogen is proteolytically activated by urokinase-type and tissue-type plasminogen activator [67]. Activation of plasminogen and prourokinase is an important mechanism in the initiation and the propagation of fibrinolytic activity. Surfactin at concentrations of 3–20 *μ*mol/L enhanced activation of prourokinase and led to conformational change in the plasminogen that further increased fibrinolysis* in vitro* and* in vivo *[74]. In a rat pulmonary embolism model, Surfactin C increased the lysis of plasma clot, when injected in the combination with prourokinase [75]. Surfactin was also able to prevent platelet aggregation, lead to inhibition of additional fibrin clot formation [38], and also enhanced fibrinolysis with the facilitated diffusion of fibrinolytic agents [76]. Detergent property of Surfactin has no any role in the antiplatelet activity, but it is caused by action on downstream signaling pathways [77]. Moreover, Surfactin has advantages over other thrombolytic agents because it has fewer side effects; therefore, it has potential for long-term use [16] as a clot-bursting agent.

### 5.9. Antibiofilm and Antiadhesion Properties of Lipopeptides

Bacteria use surface adhesion and biofilm formation as the mechanism(s) for survival on the earth. Biofilm is a community of bacteria which protects its inhabitants in extreme environmental conditions [78]. Apart from their antibacterial and antiviral activities, Surfactins have also proved to be good inhibitors of microbial adhesion and biofilm formation. For example, precoating of vinyl urethral catheters by Surfactin from* Bacillus subtilis* caused a reduction in the amount of biofilm formed [79]. A lipopeptide biosurfactant from a* Bacillus circulans *strain displayed antiadhesive property against various bacteria species [80]. Surfactin possesses specific antiadhesive activity that inhibits the biofilm formation of two selected pathogenic strains of* S. aureus* and* E. coli *on polystyrene by 97% and 90%, respectively [81]. Surfactin has an anionic nature; the antiadhesive effect can be due to the electrostatic repulsion between bacteria and the molecules of Surfactin adsorbed onto the polystyrene surface [82]. Thus, it seems that Surfactin has proven to be potential as an antiadhesive compound so that this can be used to protect the surfaces from microbial contamination [82].

### 5.10. Antifungal and Antibacterial Activities of Lipopeptides

Fungal and bacterial species are the main causative agents of plant diseases that result in the drastic reduction in the crops yield leading to economic losses to the farmers. Iturin and Fengycin are the main lipopeptides having strong antifungal activities, while Surfactin has antibacterial activity [1, 83]. The involvement of antifungal lipopeptides, Iturins and Fengycins, was found to exhibit biocontrol activity against* Bacillus* strains as well as against various plant pathogens [1]. The Surfactins are powerful surface-active compounds, which show antibacterial activity but no marked fungitoxicity (with some exceptions) [1]. On other hand, the lipopeptides of the Iturin family are potent antifungal agents; thus, they can also be used as biopesticides for plant protection [83]. Recently, a new lipopeptide referred to as “Kinnurin” isolated from* Bacillus cereus* has been found to exhibit good antifungal activity [84]. Fungitoxicity of Iturins almost relies on their ability to permeate the cell membrane of the target organism [85].

Surfactins from* Bacillus circulans *were also found to be active against multidrug-resistant bacteria such as* Alcaligenes faecalis*,* Proteus vulgaris*,* Pseudomonas aeruginosa*,* Escherichia coli, *and methicillin-resistant* Staphylococcus aureus *[86]. The minimal inhibitory and minimal bactericidal concentrations of the Surfactin were found to be much lower than those of the conventional antibiotics tested alongside [86].

## 6. Conclusion and Future Perspectives

The lipopeptides are a novel class of potent versatile weapons to deal with a variety of phytopathogens. Lipopeptides have wider applications in management of plant diseases, in cosmetics, and in food preservation and as surfactants and antiparasitic, antiviral, and antitumor/cancer agents. These lipopeptides seem to be promising biopesticides in agriculture practices for replacing harmful chemical pesticide and thus they can be considered as potent alternative tools to overcome increasing chemical resistance of phytopathogens. Lipopeptides are nontoxic, biodegradable, highly stable, ecofriendly, and nonpolluting biomolecules. These properties of the lipopeptides make them more efficient biologics for use in phytosanitation, pharmaceuticals, foods, bioremediation, and so forth. Iturin and Fengycin will be novel ecologically amenable solutions to combating resistant races of phytopathogens in agriculture practices. However, producing and applying these lipopeptides at a wider scale at present seem to be great challenges that need appropriate scale-up technologies to be evolved at industrial scale.

## Figures and Tables

**Table 1:** Lipopeptides role in food safety for inhibiting the growth of phytopathogens.

Plant disease	Phytopathogen	Lipopeptide producing microorganism	Lipopeptide inhibiting the phytopathogen	Reference(s)
Damping-off bean	*Pythium ultimum *	*Bacillus subtilis* M4	Iturin/Fengycin	[24]
Gray mold disease of apple	*Botrytis cinerea *	*Bacillus subtilis* M4	Fengycin	[24]
*Arabidopsis* root infection	*Pseudomonas syringae *	*Bacillus subtilis *6051	Surfactin	[25]
Powdery mildew of cucurbits	*Podosphaera fusca *	*Bacillus subtilis *	Iturin/Fengycin	[26]
*Fusarium* head blight (FHB) in wheat, barley and ear rot in corn	Gibberella zeae (anamorph of *Fusarium graminearum*)	*Bacillus subtilis* JA; JA026	Fengycin	[27]
Sugar beet seed infection	*Rhizoctonia solani *	*Pseudomonas fluorescens* strain 96.578	Tensin	[28]
Root and foliar diseases of soybeans	*Xanthomonas axonopodis *PV. *Glycines *	*Bacillus amyloliquefaciens* KPS46	Surfactin	[29]
Sclerotinia stem rot disease	*Sclerotinia sclerotiorum *	*Bacillus amyloliquefaciens *	Surfactin/Fengycin	[30]
Rice blast	*Magnaporthe grisea *	*Chromobacterium* sp. C61	Chromobactomycin	[31]

**Table 2:** Applications of lipopeptides in medical field.

Microorganisms	Biosurfactant type	Activity/application
*Bacillus subtilis* MZ-7 and *B. amyloliquefaciens* ES-2	Surfactin	Antimicrobial and antifungal activities [32–35]; inhibition of fibrin clot formation [36]; hemolysis and formation of ion channels in lipid membranes [32]; antitumor activity against Ehrlich's ascites carcinoma cells and antiviral activity against HIV-1 [32]; high concentration of Surfactin affects the aggregation of amyloid β-peptide into fibrils, a key pathological process associated with Alzheimer's disease [37]; antifungal, antiviral, antitumor, insecticidal, and antimycoplasma activities [38].

*Bacillus subtilis*, *B. amyloliquefaciens* B128 and *B. amyloliquefaciens *PPCB004	Iturin	Antimicrobial activity and antifungal activities against profound mycosis. Effect on the morphology and membrane structure of yeast cells [32]. Increase in the electrical conductance of bimolecular lipid membranes and acting as nontoxic and nonpyrogenic immunological adjuvant [32].

*Bacillus subtilis *	Iturin and Surfactin	Both bioagents show broad hypocholesterolemic activities [39] and can act as antibiotics, antiviral, and antitumor agents; immuno-modulators; specific toxins; and enzyme inhibitors [40].
